# Macrophage Dysfunction in Autoimmune Rheumatic Diseases and Atherosclerosis

**DOI:** 10.3390/ijms23094513

**Published:** 2022-04-19

**Authors:** Elena V. Gerasimova, Tatiana V. Popkova, Daria A. Gerasimova, Tatiana V. Kirichenko

**Affiliations:** 1 Department of Systemic Rheumatic Diseases, V.A. Nasonova Research Institute of Rheumatology, Kashirskoe Shosse, 115522 Moscow, Russia; gerasimovaev@list.ru (E.V.G.); popkova@mail.ru (T.V.P.); 2 Chair of Organization and Economy of Pharmacy, Institute of Pharmacy, A.P. Nelyubina, I.M. Sechenov First Moscow State Medical University (Sechenov University), 96k1 Ave. Vernadsky, 119526 Moscow, Russia; gerasimova_d_a@staff.sechenov.ru; 3 Laboratory of Medical Genetics, Chazov National Medical Research Center of Cardiology, 15-a Cherepkovskaya Str., 121552 Moscow, Russia; 4 Laboratory of Cellular and Molecular Pathology of Cardiovascular System, A.P. Avtsyn Research Institute for Human Morphology, 3 Tsyurupa St., 117418 Moscow, Russia

**Keywords:** autoimmune rheumatic diseases, M1-like macrophages, M2-like macrophages, inflammation, atherosclerosis

## Abstract

One of the problems of modern medical science is cardiovascular pathology caused by atherosclerotic vascular lesions in patients with autoimmune rheumatic diseases (ARDs). The similarity between the mechanisms of the immunopathogenesis of ARD and chronic low-grade inflammation in atherosclerosis draws attention. According to modern concepts, chronic inflammation associated with uncontrolled activation of both innate and acquired immunity plays a fundamental role in all stages of ARDs and atherosclerotic processes. Macrophage monocytes play an important role among the numerous immune cells and mediators involved in the immunopathogenesis of both ARDs and atherosclerosis. An imbalance between M1-like and M2-like macrophages is considered one of the causes of ARDs. The study of a key pathogenetic factor in the development of autoimmune and atherosclerotic inflammation-activated monocyte/macrophages will deepen the knowledge of chronic inflammation pathogenesis.

## 1. Introduction

Autoimmune rheumatic diseases (ARDs) are immune-mediated diseases affecting connective tissues and include rheumatoid arthritis (RA), systemic lupus erythematosus (SLE), systemic sclerosis (SSc), and Sjögren’s syndrome. The high risk of untimely death in these diseases has been found to be associated with the severity of the immunoinflammatory process that leads to irreversible damage to vital organs and systems with the development of a wide spectrum of comorbidities (infections, interstitial lung disease, malignant tumors, etc.). Among them, cardiovascular diseases hold a central position [1,2,3,4,5].

The risk of cardiovascular complications in ARD patients is high, despite the advances in diagnosis and therapy of the disease and positive trends in the reduction of cardiovascular risk in ARD patients and in the general population over the past decades [1,2]. Cardiovascular diseases (CVDs) account for a third of deaths in ARD patients [6,7,8].

In ARD patients, CVDs are mostly caused by early development and accelerated progression of atherosclerotic coronary lesions [3,9,10,11,12]. The prevalence of subclinical and clinical manifestations of atherosclerosis in immunoinflammatory diseases is 30–59% [13,14,15]. Most often, CVD and its complications develop in ARD patients with low or moderate cardiovascular risk, but with high clinical activity of the disease. In particular, it was shown in the longitudinal study that the progression of subclinical carotid atherosclerosis was more pronounced in the group of patients with active disease according to the modified Disease Activity Score-28 that included CRP level [16]. Moreover, it is currently unknown which factors play a major role in the development of severe atherosclerosis in ARD patients. In general, a combination of traditional and non-traditional risk factors, including dyslipidemia and inflammation, contributes to the development of CVD in autoimmune diseases [5].

According to modern concepts, chronic inflammation, which develops due to uncontrolled activation of both innate and acquired immunity, plays a fundamental role in all stages of ARDs and atherosclerotic processes, and can cause the development of CVD and higher mortality from cardiovascular complications [9,17]. The suggested immunopathological processes underlying chronic inflammation are the same for ARDs and atherosclerosis [17,18]:

- The systemic effect of proinflammatory cytokines: interleukin (IL)-1β, IL-6, tumor necrosis factor (TNF)-α, and interferon (IFN)-γ;
- Increased adhesion of activated neutrophils, monocytes, and platelets to the vascular endothelium under the influence of neutrophil chemokine (C-X-C motif) ligand 8 (CXCL8) or IL-8 and monocyte chemokine (C-C motif) ligand 2 (CCL2) or monocyte chemoattractant protein 1 (MCP-1);
- Further activation of platelets by neutrophils/monocytes via proteinase-activated receptors (PARs) 1 and 4 and anticitrullinated protein antibodies (ACPAs);
- Activation of vascular endothelial PAR-1 by adherent neutrophils/macrophages, exacerbating systemic inflammation and endothelial dysfunction;
- Chronic low-grade inflammation contributing to proatherogenic oxidized low-density lipoprotein (ox-LDL) modification;
- Neutrophil effect on activated platelets with the intravascular formation of a neutrophil extracellular trap (NET), which maintains intravascular proinflammatory potential through the expression of tissue factor, endothelium-activating proteases, and histones.

Modern studies of cellular and molecular markers of inflammatory and anti-inflammatory processes common to ARDs and atherosclerosis, in particular, functional disorders of macrophages, are aimed to specify the pathogenesis of these diseases and determine their clinical significance in patients with ARDs. This review attempted to determine the current status of the databases PubMed and Scopus (until January 2022) to highlight current ideas on the potential role of macrophage dysfunction in the inflammatory mechanisms of various ARDs and atherosclerosis.

## 2. Macrophage Polarization

Various types of macrophages are involved in the development of autoimmune inflammation in ARD. These resident cell types remain relatively quiescent in the healthy tissue and become activated after antigen damage, along with infiltrating monocytes/macrophages recruited as a result of proinflammatory signaling [19]. Macrophages become activated in response to endogenous and exogenous stimuli. In particular, macrophages can be activated to the proinflammatory phenotype by the microbial component lipopolysaccharide; in response to interferons (IFNs), toll-like receptor (TLR) engagement, or IL-4/IL-13 signaling, macrophages undergo M1-like or M2-like activation [20,21]. Macrophage activation is accompanied by a significant change in the gene expression profile and the formation of a cellular phenotype specific for each type of stimulus. Historically, two types of activated macrophages were first discovered. By analogy with Th (helper)1/Th2 cells, they were called M1-like and M2-like macrophages. Depending on the pathway of macrophage activation, these cells are divided into two types: classically activated type I macrophages (M1-like) (proinflammatory phenotype) and alternatively activate anti-inflammatory macrophages (M2-like) (immunomodulatory and tissue remodulating phenotype) [22,23].

The main functions of M1-like macrophages are pathogen elimination and induction of inflammatory response by secretion of proinflammatory mediators. M1-like macrophages express receptors to IL-1, TLR, and co-stimulatory molecules, thus providing induction of the inflammatory response [24]. M1-like macrophages produce proinflammatory cytokines (IL-1, IL-6, TNF-α, IL-12, IL-23, and IL-13) and cytotoxic molecules (reactive oxygen species and nitrogen metabolites). They can also be repolarized by Th2 or Th1 cytokines [25,26]. M1-like macrophages are known to synthesize not only the key cytokine of the cell-mediated immune response IL-12, but also the anti-inflammatory cytokine IL-10 [27]. M1-like macrophages are characterized by a high IL-12/IL-10 ratio [28]. The described reparative properties of M1-like macrophages are associated with the secretion of vascular endothelial growth factor (VEGF), which stimulates angiogenesis and granulation tissue formation in case of damage [29].

Alternative activation of M2-like macrophages is carried out by their stimulation by IL, glucocorticoids, immune complexes, TLR agonists, etc., contained in particular in exosomes derived from mesenchymal stem cells [30]. M2-like macrophages have a more pronounced capacity for phagocytosis compared to M1-like macrophages. M2-like macrophages express a greater number of receptors for phagocytosis, such as: CD36, a scavenger receptor for apoptotic cells; CD206, a mannose receptor; CD301, a receptor for galactose and N-acetylglucosamine residues; and CD163, a receptor for the hemoglobin/haptoglobin complex [27]. M2-like macrophages induce Th-2 cytokines (IL-4, IL-10, and IL-13), chemokine CCL18, and stimulate proliferation and angiogenesis processes [25]. M2-like macrophages are characterized by a low IL-12/IL-10 ratio [27].

## 3. Macrophage Dysfunction in Atherosclerosis

The role of macrophages in the progression of atherosclerotic vascular lesions is the most studied [31,32]. Macrophages in atherosclerotic vascular diseases play a central role in the development of plaques. Macrophages, presumably the M1-like phenotype, can induce recruitment and activation of additional macrophages, T and B cells, and dendritic cells, thereby supporting inflammation and progression of the atherosclerotic plaque. Intravascular lipid accumulation leads to recruitment of monocytes in the area of atherosclerosis development, their differentiation into macrophages, followed by metabolic reprogramming of macrophages due to atherogenic stimuli in the plaque microenvironment, such as modified lipoproteins, hypoxia, and damage-associated molecular patterns. The upregulation of anabolic pathways such as glycolysis, the pentose/phosphate pathway, and fatty acid synthesis, which appear to facilitate atherogenesis, is a general hallmark of activated immune myeloid cells in the focus of atherosclerotic plaque formation [33].

On the other hand, M2-like macrophages secrete anti-inflammatory and profibrotic mediators and limit inflammation, thus inhibiting the progression of atherosclerosis [33]. Early regression of atherosclerosis is caused by increased apoptosis of cholesterol-laden macrophages and subsequent uptake of these cells by neighboring macrophages [34]. A recently discovered activator protein (transcription factor MafB) promotes anti-inflammatory M2-like macrophage polarization and cholesterol efflux in macrophages [35]. Hyperexpression of signal transducer and activator of transcription 6 (STAT6) in vitro can also activate M2-type macrophage polarization [36]. There is evidence that STAT6-dependent polarization of macrophages to the M2 state leads to suppression of atherosclerotic inflammation and plaque regression by newly recruited Ly6Chi monocytes [37].

M1-like and M2-like macrophages have different effects on atherogenesis. M2 macrophages have a greater effect on fatty acid oxidation, while M1-like macrophages increase glycolysis [38]. M1-like macrophages predominate in the unstable atherosclerotic plaques, whereas M2-like macrophages predominate in the collagen-rich fibrous part of the plaque. This indicates that atherosclerotic plaque instability may be caused by an imbalance between M1-like and M2-like macrophages [39].

Further research should be undertaken to identify regulators of macrophage phenotype and function and to reconcile how divergent macrophage phenotypes (i.e., M1, M2) contribute to atherosclerotic plaque stability. Understanding the basis of metabolic and epigenetic reprogramming of macrophage polarization is expected to lead to the development of new therapeutic options to promote regression of the atherosclerotic process and reduce the residual inflammatory risk [31].

Meanwhile, M1-like and M2-like macrophages play essential roles in the pathology of multiple diseases in tumor growth, infectious diseases, obesity, insulin resistance, and autoimmune disorders [40,41].

## 4. Macrophage Dysfunction and Autoimmune Rheumatic Diseases

Numerous experimental data indicate the role of M1/M2 macrophage dysregulation in the development of autoimmune inflammation [42]. Classically activated M1 macrophages are induced by IFN-γ, lipopolysaccharide (LPS), granulocyte/monocyte colony-stimulating factor (GM-CSF), and TNF-α, whereas alternatively activated M2-like macrophages are induced by IL-4, IL-10, IL-13, M-CSF, immune complexes, and glucocorticoids [27,30]. Possible mechanisms of M1/M2 macrophage dysregulation in various ARDs are being studied.

### 4.1. Macrophage M1/M2 Polarization in Rheumatoid Arthritis

**Figure 1:**
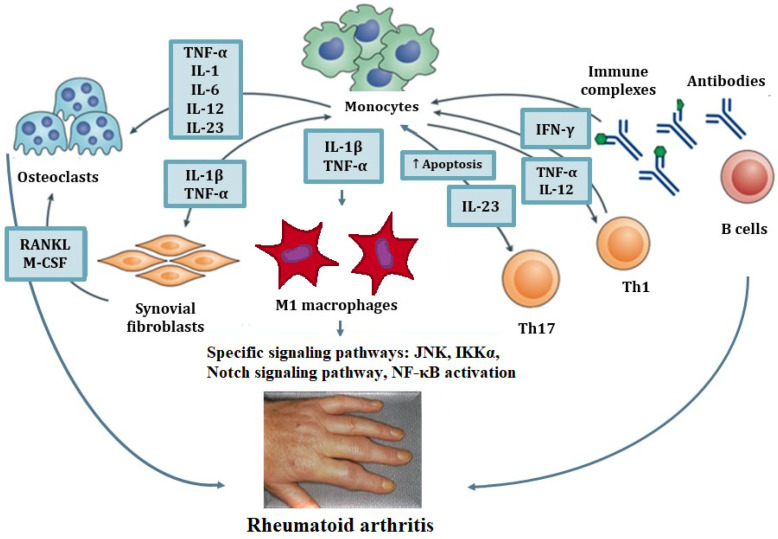
Involvement of macrophages in the development of rheumatoid arthritis. Macrophages produce cytokines that support inflammation by recruiting new immune cells (monocytes), polarizing T cells, and activating fibroblasts. Activated fibroblasts secrete receptor activator of nuclear factor kappa-B (NF-κB) ligand (RANKL) and macrophage colony-stimulating factor 1 (M-CSF), which induce osteoclast differentiation, which is enhanced by TNF and other cytokines. The formed autoimmune complexes, in turn, activate macrophages. Macrophages are also affected by cytokines produced by T cells, fibroblasts, and innate immune cells.

Macrophages in RA are predominantly of the M1-like phenotype, which contributes to RA progression by releasing various inflammatory cytokines (TNF-α, IL-1, IL-6, IL-12, and IL-23) in the synovial tissue of affected joints [43]. Macrophages produce cytokines that support inflammation by recruiting new immune cells (monocytes), polarizing T cells, and activating fibroblasts. Activated fibroblasts secrete receptor activator of nuclear factor kappa-B (NF-κB) ligand (RANKL) and macrophage colony-stimulating factor 1 (M-CSF), which induce osteoclast differentiation, which is enhanced by TNF and other cytokines. The formed autoimmune complexes, in turn, activate macrophages. Macrophages are also affected by cytokines produced by T cells, fibroblasts, and innate immune cells (Figure 1).

**Figure 2:**
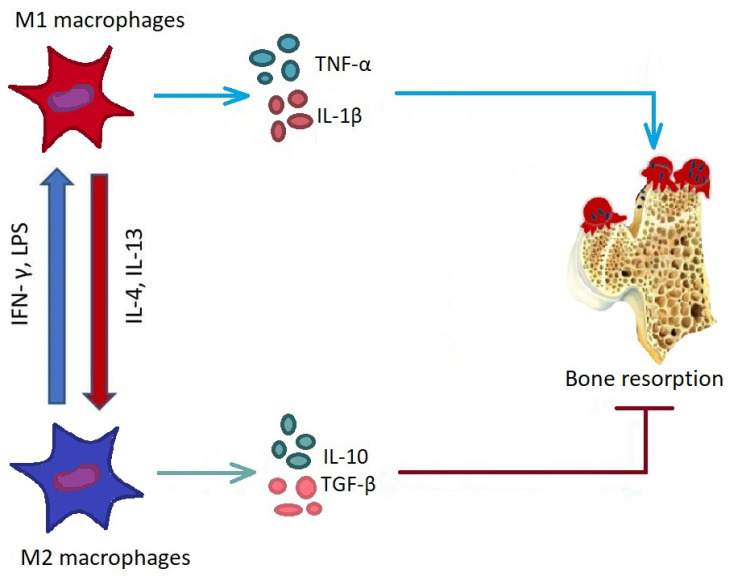
Effect of macrophages on bone resorption. M1-like macrophages can secrete TNF-α and IL-1β, inducing bone resorption. M1-like macrophages can differentiate into osteoclasts and participate in the formation of bone erosions. M2-like macrophages can secrete IL-10 and transforming growth factor-beta (TGF-β), inhibiting bone resorption.

The activity and expression level of IL-23 and SIRT proteins that modulate gene expression and is involved in the regulation of proinflammatory cytokines in RA patients was found to be impaired alongside an increase in apoptosis of peripheral blood mononuclear cells [44]. Monocytes in RA were shown to be able to penetrate the synovium and be activated to release cytokines, autoantibodies, and matrix metalloproteinase (MMP), leading to bone and cartilage destruction [45]. Figure 2 demonstrates that M1-like macrophages induce bone resorption and participate in the formation of bone erosion, while M2-like macrophages can secrete IL-10 and transforming growth factor-beta (TGF-β), inhibiting bone resorption [45].

Moreover, the involvement of M1-like and M2-like macrophages in the pathogenesis of RA is associated with their regulation of specific signaling pathways (c-Jun N-terminal kinase (JNK), IκB kinase alpha (IKKα), Notch signaling pathway) and with the activation of NF-κB [46]. It should be noted that there is a disequilibrium in the subsets of synovial macrophages of RA patients: the M1/M2 ratio is higher in patients with RA compared to healthy donors [47].

Levels of cytokines and their receptors (receptor antagonist IL-1β, IL-6, IL-1, TNF-α, IFN-γ, eotaxin, GM-CSF, M-CSF), chemokines (monocyte chemoattractant protein 1 (MCP1), and macrophage inflammatory protein 1α (MIP-1α)) were elevated in the blood of RA patients even before the development of the disease in contrast to healthy individuals and were the highest in ACPA and rheumatoid factor (RF)-positive patients [48]. Autoantibodies are very important for ARD diagnosis because of their ability to predict the severity of the disease [49]. The protective effect of ACPAs on the formation of proinflammatory M1-like macrophages was revealed by activating interferon regulatory factor 5 (IRF5) [50]. Kang et al. [51] showed that IFN-γ can stimulate macrophage polarization in the M1 phenotype. The imbalance of the M1/M2 ratio seems to be related to the number of osteoclasts (OCs) in ACPA-positive RA patients. Along with ACPAs, erythrocyte sedimentation rate (ESR) and C-reactive protein were found to correlate with the M1/M2 ratio. Thus, the M1/M2 ratio was the only significant factor affecting the number of OCs [47]. After exposure of macrophages isolated from the blood of RA patients to ACPA, the interaction between CD147 and integrin beta-1 (ITGB1) was enhanced in these cells, leading to activation of the downstream Akt/NF-κB signaling pathway and subsequent induction of NLR family pyrin domain-containing 3 (NLRP3) and expression of pro-IL-1β. In addition, ACPA can activate pannexin channels, leading to adenosine triphosphate (ATP) secretion and subsequent activation of NLRP3 inflammasome [52].

On the contrary, induction of the CD163 gene in macrophages during inflammation can lead to the preferential formation of the anti-inflammatory M2 phenotype in vitro [53]. Activated macrophages are believed to be able to affect the polarization of T-helper CD4 cells towards Th1/Th17 and vice versa. CD4 + T-effector cells can activate monocytes, and CD4 + T regulatory leukocytes can have an immunomodulatory effect on these cells, thereby inducing their anti-inflammatory properties [54]. There are data on the effect of less known proteins on the differentiation of macrophages. In particular, it has been shown that semaphorin 3A (Sema3A), a protein capable of stimulating osteoblasts, can promote IL-4-induced polarization of M2-like macrophages in vitro [55]. In vivo studies (in a mouse model) have demonstrated that administration of Sema3A reduces articular tissue damage and the severity of experimental arthritis [56]. In another experimental study, peptidyl-prolyl isomerase cyclophilin-A (CypA) promoted macrophage polarization in the proinflammatory M1 phenotype by NF-κB activating transcription, which exacerbated collagen-induced arthritis [57]. Monocytes from healthy controls, patients with RA and SLE that differentiated into monocyte-derived macrophages in the presence of circulating microparticles immune complexes (MP-IC) showed a proinflammatory (M1) profile, which was more evident using MP-IC from patients with RA than from patients with SLE [58].

### 4.2. Macrophage Dysfunction in Systemic Lupus Erythematosus

Macrophages can play different roles in pathological processes in SLE patients, often counteracting each other [59]. The role of M1-like inflammatory macrophages in SLE development is reported in many articles [60,61]. A number of researchers have demonstrated the relationship between monocyte/macrophage dysfunction and SLE activity. In particular, M1-associated genes were far more frequent in data sets from active versus inactive SLE patients [60]. Although both M1-like and M2-like macrophages contribute to the pathogenesis of lupus nephritis, several studies suggest that the M2 phenotype is the dominant subpopulation. It was shown in the study using immunohistochemical analysis of renal biopsies that M2c-like CD163+/CD68+ cells dominated in all classes of lupus nephritis [62]. In lupus-prone mice with spontaneous chronic glomerulonephritis, M2-like macrophages played the most important pathogenic role and correlated with proteinuria status [63]. In mouse SLE models, short-term ischemia/reperfusion injury of convoluted tubule epithelial cells has been shown to induce colony-stimulating factor 1 (CSF-1) production and cause an M1/M2 macrophage imbalance with a predominance of proinflammatory phenotype (M1-like) in lupus-resistant mice (MRL-Faslpr) and M2-like phenotype in lupus-susceptible mice (Sle 123), resulting in impaired tissue regeneration and accelerating the progression of lupus nephritis [64].

Schaper et al. [65] demonstrated that monocytes from peripheral blood of SLE patients have lower expression of CD163 expression and higher mRNA of IL-6 and IL-10, and differentiation of M2-like phenotype towards an M1-like phenotype reduces phagocytosis of apoptotic cells. Mediators secreted by activated macrophages, such as cytokines and a protein from the group of nuclear nonhistone proteins 1, can distort macrophage polarization towards the proinflammatory phenotype and reduce the phagocytosis of apoptotic cells underlying the pathogenesis of SLE. In the ex vivo research of macrophage changes in SLE patients caused by apoptotic cells, one of the possible mechanisms of disease pathogenesis is defective macrophage efferocytosis [66]. MP-IC from patients with systemic autoimmune diseases promotes polarization of macrophage proinflammatory differentiation by monocyte-derived macrophage microparticles into a proinflammatory profile that stimulates T-cell activation and additionally induced B-cell activation and survival. Thus, the effect of MP-IC in mononuclear phagocytes may be an important factor in modulating adaptive responses in SLE [67]. Understanding this role is of great importance because a deep knowledge of the relationship between macrophages and SLE could elucidate its pathogenesis and lay the development of macrophage-targeted therapeutic approaches [68].

### 4.3. Macrophage Dysfunction in Systemic Sclerosis

SSc is characterized by obliterative vasculopathy and tissue fibrosis. Peripheral vascular and arterial access is poor in SSc, and the vasculature is fibrosed [69]. Microvascular lesions in SSc, including endothelial damage and migration of smooth muscle cells into the vessel intima, have certain similarities with the atherosclerotic process.

There is strong evidence that macrophages play an important role in the pathogenesis of SSc [70]. It has been described in SS, that macrophages produce cytokines that support inflammation by engaging new immune cells (monocytes, neutrophils), polarizing T cells, and activating fibroblasts [71,72]. It has been hypothesized that M2-like macrophages are profibrotic. Due to their potential profibrotic and proinflammatory properties, macrophages are at the core of key SSc pathogenic processes and associated manifestations. Sequencing of the transcriptome from skin biopsies of SSc patients has allowed obtaining the full volume of RNA transcripts of the cell population. M2-like macrophages underlie molecular processes in the skin of SSc patients, with subsequent activation of interferon, activation of adaptive immunity, remodeling of the extracellular matrix, and cell proliferation [73]. Thus, macrophages are potentially important sources of fibrosis-inducing cytokines such as TGF-β [74]. In mesenchymal cells, TGF-β functions as a powerful stimulator of fibrogenesis by increasing collagen synthesis, as well as its proliferation, migration, adhesion, and transdifferentiation into myofibroblasts [75]. Indeed, the number of cells positive for M2-like macrophages markers, CD163þ and CD204þ, is increased in the skin and blood of SSc patients compared to control patients [72]. Furthermore, SSc patients have been reported to have higher circulating profibrotic macrophages in their blood [76]. Recently, it was found that skin fibrosis in mice with conditional IRF8 knockout (Irf8flox/flox; Lyz2Cre/þ), specific for myeloid cells, leads to increased mRNA levels of extracellular matrix components and increased bleomycin-induced skin fibrosis. Altered regulation of IRF8 in monocytes and macrophages may be involved in SSc pathogenesis [77].

It has been shown that a higher percentage of circulating M1/M2 mixed monocytes/macrophages is associated with interstitial lung disease, systolic pulmonary artery pressure (sPAP), and positive topoisomerase antibodies in SSc [78]. Beyond the conventional M1/M2 paradigm of macrophage subpopulations, new subpopulations of macrophages have been recently described in skin and lung biopsies from SSc patients. Notably, single-cell ribonucleic acid sequencing has provided evidence for SPP1+ lung macrophages or FCGR3A+ skin macrophages in SSc. Impaired proresolving abilities of macrophages, such as efferocytosis, may also be involved in inflammatory and autoimmune processes in SSc [79].

### 4.4. Macrophage and Sjögren’s Syndrome

There are very few studies on the role of macrophages in the development of Sjögren’s syndrome. Aota et al. showed that increased production of CXCL9 and CXCL10 from ductal cells of lip salivary glands led to the migration of CXCR3+ macrophages [80]. There was an inverse correlation between these two parameters: the number of CXCR3+CD163+ macrophages decreased as the degree of lymphocytic infiltration increased. Although CXCR3 is expressed in all innate immune cells, CXCR3+CD163+ M2-like macrophages may contribute to anti-inflammatory functions in primary Sjögren’s syndrome lesions.

SLE and Sjögren’s syndrome, diseases with anti-Sjögren’s syndrome A (anti-SSA/Ro) autoantibodies, are associated with an upregulation of IFN and IFN-stimulated type I genes, including sialic acid-binding Ig-like lectin 1 (Siglec-1), a receptor on monocytes/macrophages [81]. Therefore, researchers have recently focused on the potential role of IFN and IFN-stimulated genes in the pathogenesis of congenital heart block (CHB) [82]. Links between IFN, IFN-stimulated genes, and the inflammatory and possibly fibrosing components of the affected fetal cardiac tissue with CHB have been identified. This positions Siglec-1-positive macrophages as an integral part of the CHB process [83].

## 5. Macrophages and Accelerated Atherosclerosis in ARDs

Accelerated atherosclerosis has been observed in ARDs [3,10,11]. The molecular mechanisms that explain the acceleration of cardiovascular disease are not well understood. The differences in the pathway of atherosclerosis progression in patients with and without ARDs remain unclear.

Innate immune cells, including macrophages, are known to produce proinflammatory cytokines and chemokines that sense lipids species such as saturated fatty acids and ox-LDL [84]. However, the contribution of these cells to the development of autoimmunity and atherosclerosis requires clarification. Defects in cellular cholesterol in the hematopoietic stem and progenitor cells (HSPCs) were found in circulating monocytes in RA [85]. It is possible that the regression of the disrupted atherosclerotic lesion observed in mice with inflammatory arthritis may be initiated by early lineage-limited changes in cholesterol metabolism. Daughter monocytes can then enter the lesion with elevated cellular cholesterol, exacerbating the formation of foam cells [86]. Deficiency of ATP-binding cassette transporters A1 and G1 macrophages has been found to increase macrophage lipid accumulation, atherosclerosis, and inflammation in atherosclerotic lesions [87]. This phenotype is much more pronounced when these transporters are removed in HSPCs. It has also been found that inflammatory arthritis enhances atherogenesis by increasing the penetration of Ly6-Chi monocytes into atherosclerotic lesions, causing an increase in macrophage loading in RA [85].

In turn, changes in lipid metabolism can affect antigen presentation and cytokine production by innate immune cells. Accumulation of cholesterol crystals in a mouse model of atherosclerosis promotes caspase-1 activation via the NLRP3 inflammasome, triggers IL-1β maturation, and induces pathogenic Th17 differentiation [88]. Stimulation of TLR4 by palmitate causes reprogramming of macrophage metabolism and inflammatory responses [89]. On the other hand, liver X receptor (LXR) expression in macrophages has a negative effect on inflammatory responses through the regulation of NF-κB signaling [90]. Polymorphisms of LXR are found in patients with SLE, and LXR deficiency in mice leads to lupus-like phenotypes [91]. LXR promotes phagocytosis by upregulating MERTK expression, which controls self-tolerance and pathogenesis of SLE, and inhibits the induction of proinflammatory genes through repression of NF-κB-dependent inflammatory pathways [92].

The results of studies in mice and humans suggest that persistent inflammation caused by RA may be a causal factor in determining the severity of atherosclerotic lesions [83,84]. Zeisbrich et al. [93] reported that macrophages from patients with RA or coronary artery disease (CAD) share a common molecular phenotype of mitochondrial hyperactivation, which is mechanistically linked to glycogen synthase kinase 3b (GSK3b) deactivation. In this study, data were obtained on the restructuring of macrophage metabolism in patients with RA and CAD, leading to unhindered oxygen consumption and, ultimately, to excessive production of tissue degrading enzymes. In macrophages from patients with RA and CAD, mitochondria consumed more oxygen, generated more ATP, and built tight interorganelle connections with the endoplasmic reticulum, forming mitochondria-associated membranes. Immunostaining of atherosclerotic plaques and synovial lesions confirmed that most macrophages had inactivated GSK3b. The underlying molecular defect relates to the deactivation of GSK3b, which controls mitochondrial fuel influx and, as such, represents a potential therapeutic target for anti-inflammatory therapy.

## 6. Antirheumatic Drugs and Macrophages

Functional disorders of macrophages and their mediators are important for understanding both the development of the disease itself and possible therapeutic interventions for ARDs [94]. The data on the effect of antirheumatic drugs on the development of atherosclerosis and its complications are of great interest to scientists. The concept of the role of macrophages in the development of subclinical inflammation formed the basis for studying the atheroprotective effect of antirheumatic drugs. Based on the CANTOS [95], CIRT [96], and COLCOT studies [97], several new anti-inflammatory and anticytokine agents are expected to be developed for the treatment of atherosclerosis [98].

A convincing “antiatherosclerotic” effect was demonstrated by the Canakinumab Anti-inflammatory Thrombosis Outcomes Study (CANTOS) on the use of a monoclonal antibody to IL-1β in patients with severe atherosclerotic vascular lesions [94]. CANTOS helped to define the inflammatory pathway from IL-1 to IL-6 to CRP as a central target for atheroprotection. IL-1β is known to be synthesized by macrophages under the influence of various pathogenic patterns (pathogen-associated molecular and damage-associated molecular patterns) that interact with membrane-like receptors (TLR and cytoplasmic nucleotide-binding oligomerization domain-like receptors).

The involvement of IL1β in atherogenesis is needed for the adherence of monocytes and leukocytes to the vascular endothelium, the vascular smooth muscle cell growth, the synthesis of inflammatory mediators, nitric oxide, and prostaglandins, and its “procoagulant” activity [99]. “Proatherogenic” factors such as NETs, cholesterol and calcium phosphate crystals, and ox-LDL in macrophages induce IL-1β synthesis by activating NLRP3 inflammasomes (nucleotide-binding domain, leucine-rich-containing family, pyrin domain-containing 3, or NOD-like receptor protein 3) [100]. There is evidence that the production of NLRP3-IL-1β may contribute to the development of accelerated atherosclerosis in clonal hematopoiesis [101].

Another study, CIRT [95], examined the risk of cardiovascular events during methotrexate therapy. The antiatherogenic effect of methotrexate is associated with the suppression of IF-γ-induced transformation of macrophages into foam cells, activation of the ATP-binding cassette transporter-A1, which is involved in reverse cholesterol transport, and a decrease in the expression of endothelial adhesion molecules [102]. Reiss et al. [103] showed on cell culture (human THP1 monocytes/macrophages) that activation of the A2A adenosine receptor by methotrexate enhances reverse cholesterol transport and reduces the transformation of “foamy” cells. The proinflammatory effects of methotrexate on secretion of cytokines IL-1, IL-6, and TNF-α have been demonstrated in human monocyte/macrophage cell cultures [104].

The success of TNF-α inhibitors therapy in RA patients may also indicate the involvement of macrophages in the development of RA [105]. Anti-TNF agents may induce alternative functions in macrophages activated in inflammatory conditions, with an inhibition of inflammatory cytokines (TNF-α, IL-6, IL-12) and an increase in phagocytosis [106]. These results were associated with increased early production of IL-10, responsible for higher STAT3-dependent control of inflammation [107]. As shown in a mouse model of colitis, the therapeutic response to anti-TNF depends on IL-10 signaling in mucosal macrophages [108]. The FcγR-mediated effect of IL-10 on the macrophage phenotype induced by anti-TNF monoclonal antibodies may be of less importance in ARDs such as RA. At the same time, there is evidence that IL-10 inhibits the expression of IL-17 and retinoid-related orphan receptor γt (RORγt) in macrophages and suppresses macrophages of the “proinflammatory” phenotype M1 [109].

Based on current advances, it seems clear that macrophage dysfunction is one of the important components of accelerated atherosclerosis in ARDs. Further research is needed to advance interdisciplinary research between the immune system and atherosclerosis to develop novel therapeutic strategies targeting autoimmune inflammation.

## 7. Conclusions

Collective evidence shows that changes in the macrophage differentiation, polarization, and activation at the sites of inflammation can play a decisive role in the pathogenesis of a wide variety of ARDs and atherosclerosis. Due to their pro- and anti-inflammatory properties, macrophages are at the intersection of the key pathogenetic processes of ARDs and atherosclerosis. Considering the accelerated atherosclerosis development and increased risk of CVD in patients with ARD, further study of macrophages activation in ARD patients will clarify their role in the maintenance of autoimmune inflammation and progression of atherosclerosis in rheumatic diseases.

## Abbreviations

ACPAs	anticitrullinated protein antibodies
ARDs	autoimmune rheumatic diseases
ATP	adenosine triphosphate
CAD	coronary artery disease
CCL2	chemokine (C-C motif) ligand 2
CHB	congenital heart block
CRP	C-reactive protein
CSF-1	colony-stimulating factor 1
CTGF	connective tissue growth factor
CVCs	cardiovascular complications
CVDs	cardiovascular diseases
CVR	cardiovascular risk
CXCL8	chemokine (C-X-C motif) ligand 8
ESR	erythrocyte sedimentation rate
GM-CSF	granulocyte/monocyte colony-stimulating factor
GSK3b	glycogen synthase kinase 3b
HC	healthy controls
HSPCs	hematopoietic stem and progenitor cells
IFN-γ	interferon-gamma
IKKα	IκB kinase alpha
IL	interleukin
IRF5	interferon regulatory factor 5
ITGB1	integrin beta-1
JNK	C-Jun N-terminal kinase
LPS	lipopolysaccharide
LXR	liver X receptor
M1	classically activated macrophages
M2	alternatively activated macrophages
MCP-1	monocyte chemoattractant protein 1
M-CSF	macrophage colony-stimulating factor
MDM	monocyte-derived macrophages
MIP-1α	macrophage inflammatory protein 1 alpha
MMP	matrix metalloproteinase
MP-IC	microparticles immune complexes
NET	neutrophil extracellular trap
NF-κB	nuclear factor kappa-B
NLRP	NLR family pyrin domain-containing 3
OA	osteoarthritis
OC	osteoclast
Ox-LDL	oxidized low-density lipoprotein
PAR	proteinase-activated receptors
RA	rheumatoid arthritis
RANKL	receptor activator of nuclear factor kappa-B (NF-κB) ligand
RF	rheumatoid Factor
Sema3A	semaphorin 3A
Siglec-1	sialic acid-binding Ig-like lectin 1
SIR1	silent information regulator 1 proteins
SIRT	sirtuins
SLE	systemic lupus erythematosus
sPAP	systolic pulmonary artery pressure
SSc	systemic sclerosis
STAT6	signal transducer and activator of transcription
TGF-β	transforming growth factor-beta
Th	T-helper
TLR	toll-like receptor
TNF-α	tumoral necrosis factor-alpha
VEGF	vascular endothelial growth factor
